# AdenoBuilder: A platform for the modular assembly of recombinant adenoviruses

**DOI:** 10.1016/j.xpro.2022.101123

**Published:** 2022-01-20

**Authors:** Yumi Jang, Fred Bunz

**Affiliations:** 1 Department of Radiation Oncology and Molecular Radiation Sciences, Kimmel Cancer Center at Johns Hopkins, Baltimore, MD 21287, USA

**Keywords:** Microbiology, Molecular Biology, Biotechnology and bioengineering

## Abstract

The AdenoBuilder platform enables the *in vitro* assembly of recombinant vectors from plasmid inserts that span the adenovirus genome. Two advantages of AdenoBuilder are the ease of modifying the genome and the ability to produce multicomponent vectors in a single step, facilitating parallel approaches to vector optimization. This protocol describes how to introduce transgenes in place of the endogenous Human Adenovirus serotype 5 (HAd5) E1 and/or E3 genes and can be applied to other parts of the HAd5 genome.

For complete details on the use and execution of this protocol, please refer to Miciak et al. (2018).

## Highlights

• *In vitro* assembly of synthetic adenovirus genomes from plasmid components
• Simplified construction of adenovirus serotype 5 (Ad5)-based vectors
• AdenoBuilder facilitates combinatorial approaches to vector optimization
• Infectious virus produced 5 days after genome assembly

## Graphical abstract

**Figure:**
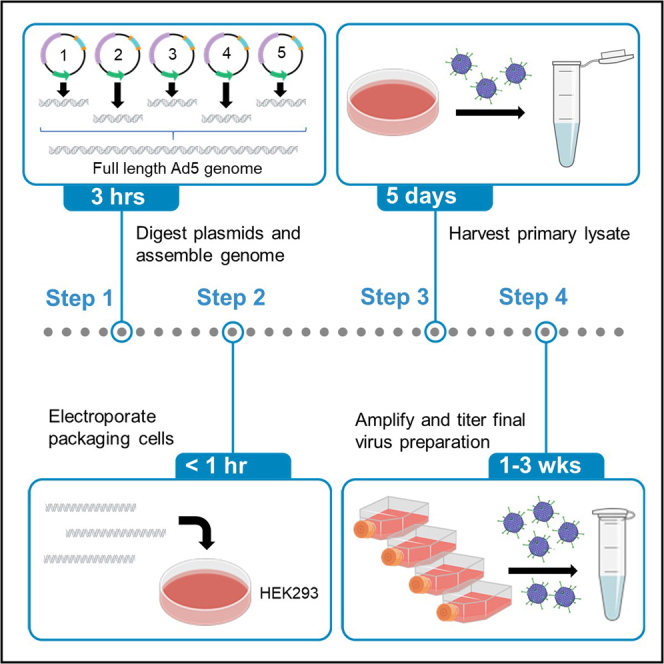

## Before you begin

With the AdenoBuilder system, a researcher can rapidly and efficiently modify any desired genomic region, or multiple regions, in the Human Adenovirus serotype 5 (HAd5) genome and rescue infectious viruses. This protocol describes the specific steps for introducing transgenes in place of the endogenous HAd5 E1 and/or E3 genes, a common goal of many projects. We have also used this protocol to introduce functional point mutations and heterologous epitopes into other parts of the HAd5 genome (Miciak et al., 2018).

**Figure 1:**
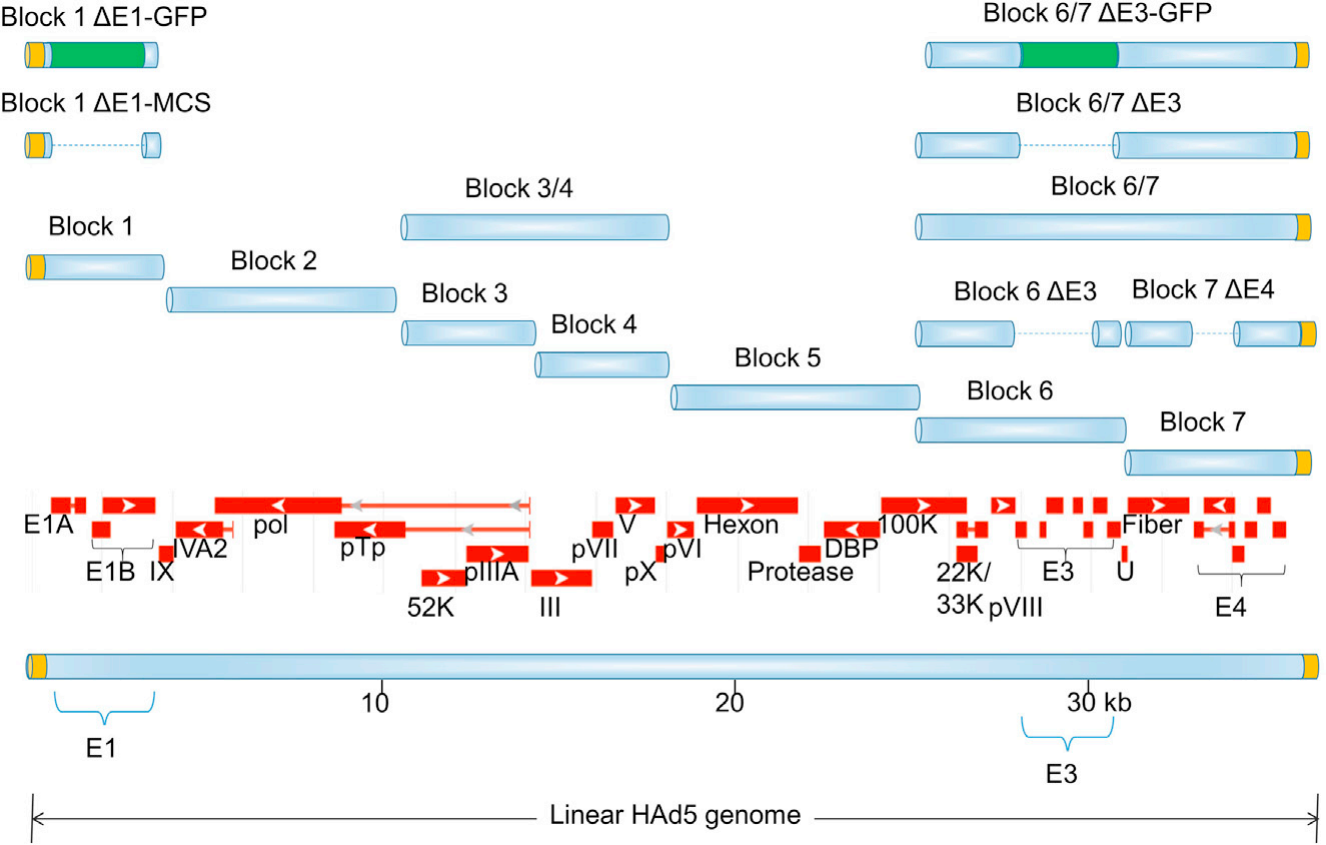
The expanded AdenoBuilder system Functional adenovirus genomes are comprised of seven segments, individually cloned into Blocks 1–7. Several of these segments have been consolidated into larger plasmids, as shown, to facilitate a simplified 5-piece assembly. Blocks that harbor deletions (dotted lines) can be used in combination to accommodate transgenes up to 8 kb in size. GFP expression cassettes, useful for functional titering, are shown in green; endogenous HAd5 viral genes and gene clusters are indicated in red; inverted terminal repeats (ITRs) are shown in orange.

The AdenoBuilder system is modular, consisting of a series of seven cloned DNA segments that span the 36 kb HAd5 genome (Figure 1). To engineer a new recombinant HAd5 genome, the researcher must first obtain the required plasmid modules, or “blocks”, that compose the desired configuration of the final vector. The first version of the AdenoBuilder system consisted of seven wild type blocks (Blocks 1–7) and three modified blocks designed to accommodate transgenic elements of various sizes (Miciak et al., 2018). Here, the inserts from Blocks 3 and 4 and Blocks 6 and 7 have been consolidated into two plasmids, respectively designated Block 3/4 and Block 6/7. These larger blocks facilitate a highly efficient 5-piece assembly that is suitable for most simple vector designs.

Many investigators prefer to work with HAd5 mutants that lack genes in the E3 region (Ginsberg et al., 1989). The E3 genes are not essential for virus replication. Because these genes function to suppress the adaptive immune responses in the host, E3 deletions are a useful safety feature that is particularly suitable for cell-based studies. The entire E3 region is deleted in Block 6 ΔE3 and Block 6/7 ΔE3 to facilitate the assembly of such vectors. Investigators who wish to retain some of the functions of E3 can easily customize Block 6.

User-selected blocks are seamlessly joined by a modified Gibson assembly procedure (Miciak et al., 2018). BstBI-digested plasmid DNAs are processed by the heat-labile T5 exonuclease, which creates an extended 3′ overhang at both ends. The complementary strands from adjacent blocks can then anneal. The ends are trimmed to remove mismatches, filled in and ligated.

Each of the seven segments must be represented in the vector design. Another important consideration is the predicted size of the final assembly. HAd5 can encapsidate recombinant genomes ranging from 34 to 38 kb in size. Attempts to package genomes outside this size range may result in significantly reduced viral titers (Bett et al., 1993).

We have created two new blocks, Block 1 ΔE1-GFP and Block 6/7 ΔE3-GFP, that incorporate a Green Fluorescent Protein (GFP) expression cassette into E1 and E3, respectively (Figure 1). These GFP cassettes can be used as “stuffer” sequences, to increase the size of a planned genome assembly and thereby facilitate optimal packaging. Following packaging and amplification, the expression of GFP serves as a useful measure of functional viral titer. GFP-positive cells can be quantified by fluorescence microscopy or flow cytometry.

### Design a viral vector genome

**Timing: 1 day**

1. Transgenes are most conveniently inserted into the plasmids that harbor deletions in E1 (Block 1 ΔE1-MCS) or E3 (Block 6 ΔE3, Block 6/7 ΔE3 or Block 6/7 ΔE3-GFP). A polylinker in Block 1 ΔE1-MCS facilitates directional cloning (Figure 2). The deletions in Block 6/7 ΔE3 and Block 6/7 ΔE3-GFP are flanked by BamHI and SalI sites. Additional details regarding cloning into these vectors are provided in Miciak et al. (2018). Complete plasmid sequences can be directly downloaded from the Addgene website.

**Figure 2:**
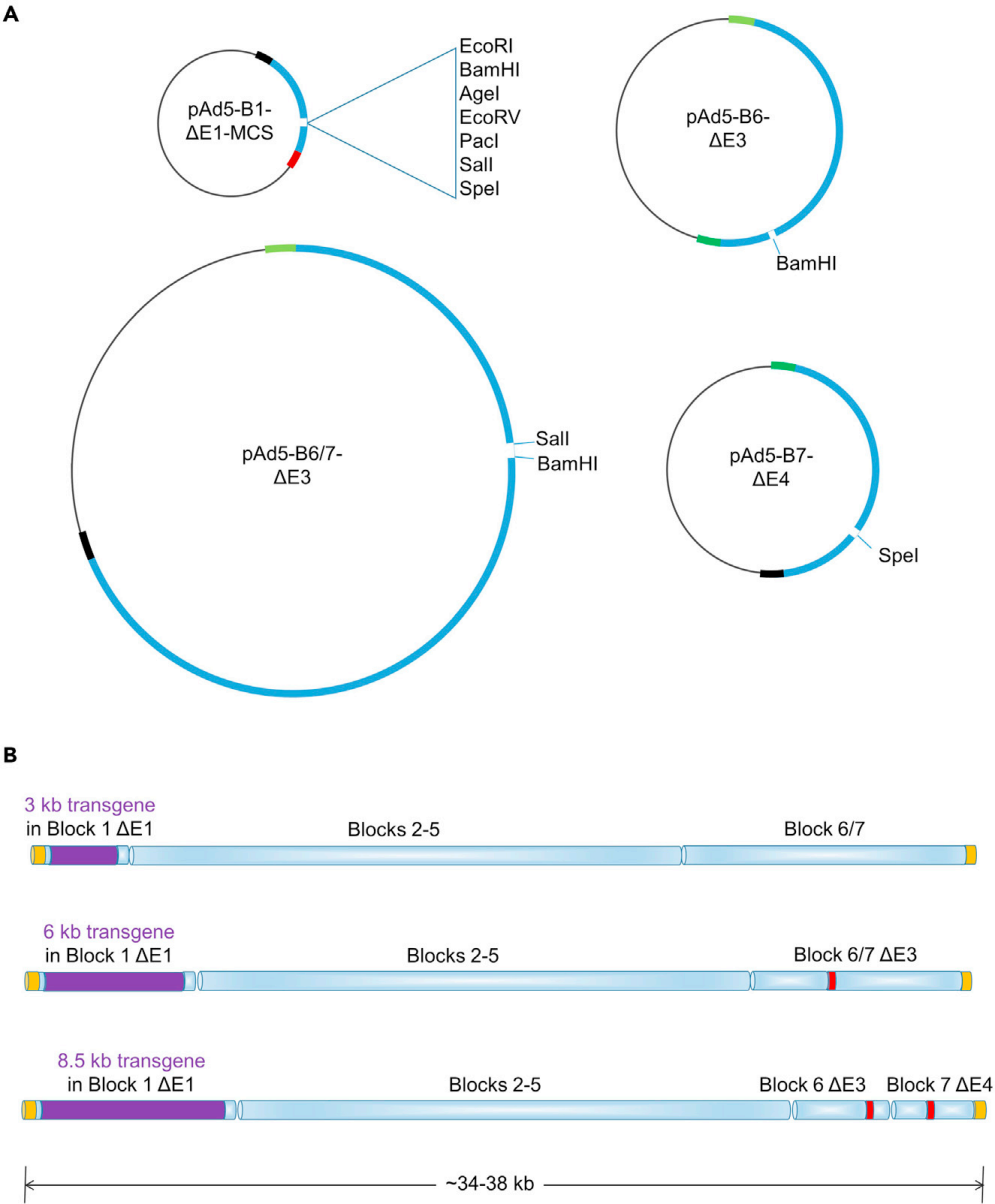
AdenoBuilder blocks for transgene incorporation (A) The AdenoBuilder plasmid components pAd5-B1ΔE1-MCS, pAd5-B6ΔE3, or pAd5-B6/7ΔE3 can be readily modified to accommodate DNA cassettes of various sizes. pAd5-B7ΔE4 can be employed in place of pAd5-B7 to create additional capacity for large transgenes. (B) Used in various combinations, these plasmids allow the customized assembly of recombinant viruses capable of delivering transgenic elements up to 8.5 kb in size. Transgenes (purple bars) can be inserted in place of viral E1, as shown, and/or viral E3. The positions of E3 and E4 deletions are indicated in red. Figure modified from Miciak et al. (2018).
2. Viruses that retain the wild type E1 region will be replication competent.
3. Viruses lacking E3 genes may have reduced pathogenicity (Ginsberg et al., 1989).
4. Block 1 ΔE1-MCS, Block 6 ΔE3 and Block 7 ΔE4 can be used individually or together to accommodate large transgenes, or more than one transgene cassette (Figure 2).
5. Any block modification must preserve the ends defined by the BstBI sites. The short sequence overlaps shared by adjacent blocks are required for assembly.

### Select and procure a panel of plasmids for modification and assembly

**Timing: 1 hto1 week**

6. Each of the seven segments must be included in the final assembly.
7. Most common designs will incorporate Block 3/4 and Block 6/7, or one of its derivatives. These blocks contain two segments each, and thus enable a 5-piece assembly (Figure 1).
8. Investigators who wish to introduce alterations within the regions spanned by Block 3/4 or Block 6/7 may opt to design a 6- or 7- piece assembly that incorporates the single-segment blocks (Blocks 3, 4, 6 and/or 7).
9. The size of the final assembly is calculated by adding the sizes of each of the Blocks (including deletions) and the size of any customized features (e.g., transgene expression cassettes). The total size must fall into the ∼ 34–38 kb range. Blocks containing GFP expression cassettes can be used to adjust the size of the final design. Useful information regarding plasmids and their inserts are provided in the table below.
10. All of the plasmids described in this protocol are available as bacterial stabs from Addgene. We recommend the PureLink Midiprep kit for plasmid purification, although other commercially available kits should also be suitable.
11. Plasmid stabs obtained from Addgene are in the Stable bacterial strain (NEB). We have found that these plasmids can be routinely propagated in DH5α cells as well.
12. The concentration and purity of each plasmid are assessed by spectrophotometry. The OD 260/280 nm ratio should be between 1.7 and 1.9. We recommend the NanoDrop spectrophotometer for this purpose.

***Note:*** Addgene now offers the complete set of plasmids as a single toolkit, at a reduced cost. Alternatively, each of the AdenoBuilder plasmids can be acquired separately. Preparation of the plasmid pAd5-B1 typically results in a lower DNA yield compared to the other AdenoBuilder plasmids. This is normal and not usually a problem. If desired, bacteria transformed by pAd5-B1 can be grown in a larger culture volume, as for a low copy plasmid.

**CRITICAL:** The pJET1.2 vector backbone present in each of the vectors is not integrated into genome assemblies. All size calculations are based on insert sequences only.

## Key resources table

**Table:** 

REAGENT or RESOURCE	SOURCE	IDENTIFIER
**Antibodies**		

Adenovirus Type 5 Hexon Polyclonal Antibody, FITC (1:100 dilution)	Invitrogen	PA1-73053

**Bacterial and virus strains**		

NEB 5-alpha (DH5α) Competent E. coli	New England Biolabs	C2987
Ad5 reference material	ATCC	VR-1516

**Chemicals, peptides, and recombinant proteins**		

DMEM cell culture medium, high glucose, pyruvate	Gibco	11995073
Corning Fetal Bovine Serum, 500 mL, Regular	Corning	35-010-CV
Penicillin Streptomycin	Gibco	15140122
BstBI enzyme	New England Biolabs	R0519S
NEBuilder HiFi DNA Assembly Master Mix	New England Biolabs	E2621S
PBS pH7.4 (1×)	Gibco	10010023
Trypsin-EDTA (0.05%), phenol red	Gibco	25300062
Bovine Serum Albumin (BSA)	MilliporeSigma	A9647
Absolute Ethanol (200 proof)	Thermo Fisher Scientific	T038181000
Ammonium acetate solution (7.5 M)	MilliporeSigma	A2706-100ML
UltraPure Distilled Water	Invitrogen	10977-015
Paraformaldehyde solution 4% in PBS	Santa Cruz Biotech	sc-281692
Triton X-100, BioXtra	MilliporeSigma	T9284
Lipofectamine 3000 Transfection Reagent	Thermo Fisher Scientific	L3000001

**Critical commercial assays**		

QuickTiter Adenovirus Titer Immunoassay Kit	Cell Biolabs	VPK-109
PureLink HiPure Plasmid Filter Midiprep Kit	Invitrogen	K210014
SF Cell Line 4D-Nucleofector X Kit L	Lonza	V4XC-2012

**Experimental models: Cell lines**		

Human embryonic kidney 293 (HEK293) cells	ATCC	CRL-1573

**Oligonucleotides**		

pJET1.2-forward (5′-CGACTCACTATAGGGAGAG CGGC-3′)	Thermo Fisher Scientific	SO501
pJET1.2-reverse (5′-AAGAACATCGATTTTCCA TGGCAG-3′)	Thermo Fisher Scientific	SO511

**Recombinant DNA**		

AdenoBuilder toolkit	This paper	Addgene; #1000000176
pAd5-B1	Miciak et al. (2018)	Addgene;#12255
pAd5-B2	Miciak et al. (2018)	Addgene; #122552
pAd5-B3	Miciak et al. (2018)	Addgene; #122553
pAd5-B4	Miciak et al. (2018)	Addgene; #122554
pAd5-B5	Miciak et al. (2018)	Addgene; #122555
pAd5-B6	Miciak et al. (2018)	Addgene; #122556
pAd5-B7	Miciak et al. (2018)	Addgene: #122557
pAd5-B1 ΔE1-MCS	Miciak et al. (2018)	Addgene; #122558
pAd5-B6ΔE3	Miciak et al. (2018)	Addgene;#122559
pAd5-B7ΔE4	Miciak et al. (2018)	Addgene;#122560
pAd5-B3/4	This paper	Addgene; #175785
pAd5-B6/7	This paper	Addgene; #175747
pAd5-B6/7 ΔE3	This paper	Addgene; #175748
pAd5-B1 ΔE1-GFP	This paper	Addgene; #179203
pAd5-B6/7 ΔE3-GFP	This paper	Addgene; #179202
pAd5-B6 ΔE3-GFP	This paper	Addgene: #179201

**Software and algorithms**		

NEBiocalculator	New England Biolabs	https://nebiocalculator.neb.com/#!/dsdnaamt

**Other**		

4D-Nucleofector Core Unit	Lonza	AAF-1002B
4D-Nucleofector X Unit	Lonza	AAF-1002X
EVOS M5000 Imaging System	Thermo Fisher Scientific	AMF5000
NanoDrop One/One spectrophotometer	Thermo Fisher Scientific	ND-ONE-W
Fyrite Classic Combustion Analyzer	Thomas Scientific	5606A13

## Materials and equipment

**Table:** AdenoBuilder plasmids

Plasmid	Total plasmid size (bp)	Segment	Insert size	Plasmid mass (μg)/500 fmol
pAd5-B1	6747	1	3759	2.09
pAd5-B2	10133	2	7145	3.13
pAd5-B3	6626	3	3638	2.05
pAd5-B4	6532	4	3544	2.02
pAd5-B5	10053	5	7065	3.11
pAd5-B6	8853	6	5865	2.74
pAd5-B7	8039	7	5050	2.48
pAd5-B1 ΔE1-MCS	3742	1	754	1.55
pAd5-B6ΔE3	5908	6	2914	1.83
pAd5-B7ΔE4	5456	7	2468	1.69
pAd5-B3/4	10146	3 and 4	7167	3.14
pAd5-B6/7	13878	6 and 7	10890	4.29
pAd5-B6/7 ΔE3	10943	6 and 7	7955	3.38
pAd5-B1 ΔE1-GFP	5660	1	2672	1.75
pAd5-B6 ΔE3-GFP	5911	6	2923	1.83
pAd5-B6/7 ΔE3-GFP	12877	6 and 7	9889	3.98

Plasmid DNAs are stored at −20°C, and are stable for at least 1 year

**CRITICAL:** This method results in the production of infectious adenovirus 5. All steps following electroporation must be performed in a BSL2-certified biosafety cabinet.

***Alternatives:*** Our laboratory routinely uses the 4-D Nucleofector system to deliver plasmid assemblies to HEK293 cells. We expect that alternative equipment setups for mammalian cell electroporation would be adequate substitutes. Lipofection can be performed instead of electroporation, as noted below.

## Step-by-step method details

### Assembly of HAd5 genome

**Timing: 3 h**

Ad5 genomes are assembled *in vitro* from plasmid blocks. In most cases, a vector will be assembled from a combination of pre-made and user-customized plasmids.

1. Digest a DNA mixture containing 500 fmol of each of the plasmid DNAs.
  a. Add 500 fmol of each plasmid to a single 1.5 mL tube. The conversion of dsDNA mass units to fmol is easily performed with the NEBiocalculator.
  b. Prepare a 100 μL digestion reaction.
    i. To the pooled plasmid DNAs, add 10 μL of 10× CutSmart buffer, provided with the BstBI enzyme.
    ii. Add UltraPure distilled water to a total volume of 95 μL.
    iii. Add 5 μL of restriction enzyme BstBI (20 Units/μL).
  c. Vortex briefly, then pulse centrifuge to collect the liquid at the bottom of the tube.
  d. Incubate the reaction in a 65°C heating block or water bath for 30 min.
2. Precipitate digested plasmid DNAs.
  a. Add 25 μL of 7.5 M Ammonium Acetate and 300 μL of 100% ethanol to the 100 μL digest. Vortex briefly.
  b. Centrifuge at room temperature for 5 min at maximum speed.
  c. Decant the supernatant.
  d. Add 500 μL of 70% ethanol to wash the pelleted DNA. Centrifuge at maximum speed for 1 min at room temperature. A small pellet should be visible.
  e. Carefully decant the supernatant and use a pipette to remove remaining liquid, taking care not to disturb the DNA pellet. Air-dry the pellet at room temperature for 15 min, or until the last visible traces of ethanol have evaporated.
  f. Dissolve the DNA pellet in 5 μL of ultrapure distilled water. Incubate the capped tube at 37°C for 30 min to ensure that the pellet is completely in solution.
3. Set up the DNA Assembly reaction
  a. Add 5 μL of 2× NEBuilder HiFi DNA Assembly Master Mix to DNA, vortex briefly and centrifuge to bring all liquid to the bottom of the tube.
  b. Incubate the reaction at 45°C in a heating block or water bath for 1 h.
  c. Following incubation, put the assembled DNA on ice or store at −20°C for subsequent electroporation.

**CRITICAL:** Do not extensively dry the DNA pellet with heat or under vacuum. Removal of all moisture may make it difficult to fully dissolve the DNA. While the standard NEBuilder plasmid protocol calls for incubation at 50°C, conducting the assembly at 45°C results in increased production of full length genomes. Confirm the temperature of the water bath or heating block beforehand.

***Note:*** The total amount of DNA in the digest should range between 15 and 17 μg. It is not necessary to purify the assembled DNA before electroporation.

### Electroporation of packaging cells

**Timing: 45 min (+ 5 days)**

HEK293 cells are electroporated with the 4D-Nucleofector system equipped with the X module, using the materials provided with the SF Cell Line Kit. Use the CM130 program, as recommended for HEK293 cells. Turn on the 4D-Nucleofector System and set up the program in advance. Supplement the SF solution as per manufacturer’s instructions. Add 4 mL culture medium to a fresh T-25 flask and prewarm in the incubator.

***Note:*** HEK293 are maintained in DMEM cell culture medium supplemented with 6% fetal bovine serum and 1% penicillin-streptomycin. Approximately 2 × 10^6^ cells should be freshly seeded in a T-25 flask the day prior to electroporation so that the cells are near confluence at the time of collection. We recommend using HEK293 cells that have been continuously passaged no more than 10 times.

4. Aspirate medium. Detach HEK293 cells in 1 mL 0.05% trypsin/EDTA by incubation at 37°C for 5 min, or until they detach. Add 4 mL complete medium and gently swirl the flask to assure that all cells are detached. Count cells and transfer a calculated volume containing 2 × 10^6^ cells to a 15 mL tube.
5. Centrifuge cell suspension at 90 *g* for 10 min at room temperature. Aspirate and discard the supernatant.
6. Gently resuspend the cell pellet with 5 mL PBS at room temperature, centrifuge again, and discard the supernatant.
7. To the 1.5 mL tube containing the assembled DNA (10 μL) add 90 μL of supplemented Nucleofector SF solution.
8. Add DNA/SF solution mixture (100 μL) to the cell pellet. Resuspend cells by gently pipetting up and down and transfer to a 100 μL cuvette.
9. Gently tap the cuvette on a solid surface to dislodge any bubbles, and place into the 4D Nucleofector.
10. Electroporate the cells with the preprogrammed pulse CM130.
11. Immediately following electroporation, transfer the cells to the pre-warmed growth medium in the T25 flask. Return the flask to the incubator.

**CRITICAL:** The CO_2_ level in the cell culture incubator must be at 5% to ensure efficient virus packaging. We have found that packaging efficiency is dramatically reduced when CO_2_ levels are 3% or below. For consistent results, incubators should be calibrated regularly.

***Alternatives:*** Lipofection can be performed instead of nucleofection. We find that the titers of the primary lysates obtained after lipofection are usually lower. We recommend Lipofectamine 3000 Transfection Reagent (ThermoFisher cat# L3000001), used according to the manufacturer’s instructions, for this purpose.

### Harvesting virus

**Timing: 1 h**

**Figure 3:**
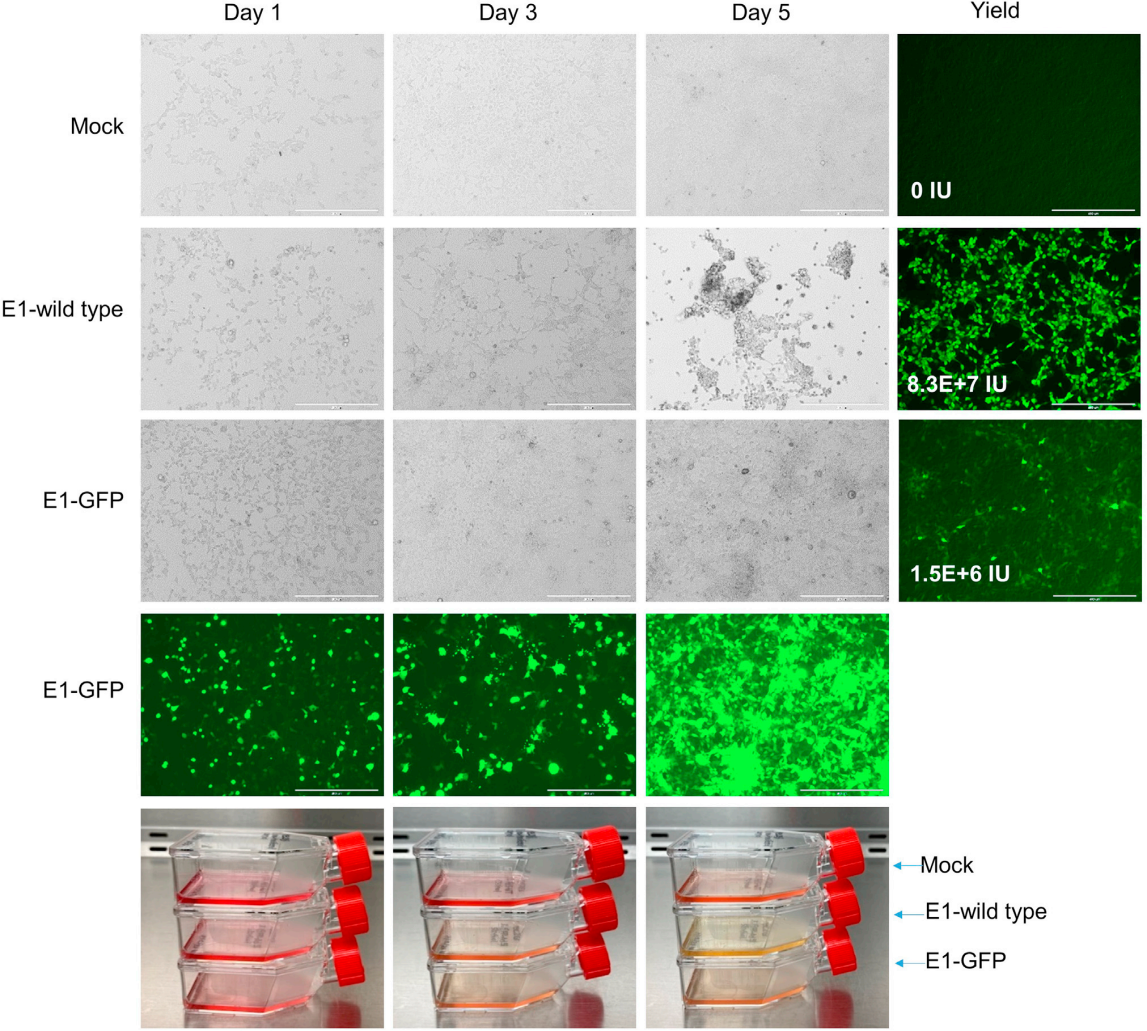
Packaging synthetic HAd5 genomes In this experiment, a wild type HAd5 genome (containing unmodified Block 1) and a recombinant genome that expresses GFP from a transgene cassette (containing Block 1 ΔE1-GFP) were assembled in parallel from six plasmids. Following electroporation, cells were transferred to a T25 flask (step 11) and monitored for 5 days. Cytopathic effects were most apparent in the wild type HAd5 flask. Despite the absence of clear cytopathic effects, the levels of GFP increased in the flask with the recombinant genome lacking adenovirus E1 (E1-GFP). Acidification of the medium, indicated by the yellow color, was most striking in the wild type HAd5 flask but was also apparent in the E1-GFP flask. Following harvest, the viral content of the primary lysates were estimated by the rapid fluorescence focus-formation assay (steps 24–36). Magnification, 10×, Scale bar = 400 μm.

Packaging cells may exhibit cytopathic effects, including significant amounts of detachment, 5 days after electroporation. If the desired virus retains wild type E1 genes, the cytopathic effects will be pronounced and the medium may acidify, turning from red to yellow (Figure 3). Cytopathic effects are typically less obvious when packaging recombinant viruses in which the E1 region has been replaced. Proceed with the harvest on day 5 regardless of the appearance of the cells. Equilibrate a dry ice/ethanol bath and a 37°C water bath in advance.

12. Without removing the medium, use a disposable cell scraper to detach the cells. Transfer all cells and culture media into a single 15 mL conical tube.
13. Vortex the cell suspension for at least 15 s and immediately freeze the cells by placing the tightly capped tube in the dry ice/ethanol bath.
14. Completely thaw the lysate in a 37°C water bath. Remove when the lysate is just thawed and vortex for at least 15 s.
15. Repeat freeze-thaw-vortex cycle two additional times.
16. Following the last thaw and vortex, centrifuge the lysate at 10,000 *g* for 10 min at 4°C.
17. Transfer the virus-containing supernatant to a new 15 mL conical tube.

***Note:*** Cells may detach completely by day 5. Proceed with step 12 regardless.

**Pause point:** The primary viral lysate can be stored at −80°C until ready for analysis or amplification.

### Viral amplification

**Timing: 3–5 days**

One or more rounds of amplification are usually required for the generation of high-titer recombinant adenovirus stocks. To amplify the primary lysate, seed approximately 5–6 × 10^6^ HEK293 cells in a T-75 flask containing 10 mL culture medium one day before infection.

18. Infect cells with 2 mL of the primary viral lysate from step 17.
19. Periodically monitor cells under the microscope for evidence of cytopathology, beginning at 3 days post infection. Handle flasks with care to avoid dislodging the cell monolayer.
20. When cytopathic effects are evident, but before the cells fully detach, gently aspirate the medium. Do not wash the monolayer.
21. Add 0.5 mL of PBS (room temperature) and collect the cells with a cell scraper. Transfer the cell suspension to a 15 mL conical tube.
22. Lyse the cells by the freeze-vortex-thaw method, as described in steps 13–16.
23. Remove the virus-containing supernatant and aliquot into screw-top cryotubes.

**Pause point:** The amplified viral lysate can be stored at −80°C until ready for analysis or further amplification.

***Note:*** Amplified viral lysates are often yellow due to acidification of the medium during packaging. This is a reliable indication of a relatively high viral titer. In this case, use 0.2 mL of the primary lysate for amplification.

***Optional:*** Amplified lysates can be used directly for many applications without further purification. If desired, a more concentrated and pure virus stock can be generated after one additional round of amplification. We recommend a simplified cesium chloride gradient centrifugation method for virus concentration and purification detailed in Luo et al. (2007).

### Estimation of viral titers

**Timing: 3 days**

Viral titers can be estimated by a simple focus-formation assay in which infected cells are fixed, permeabilized and stained with a fluorescent anti-hexon antibody. Plate HEK293 cells in a 48-well cell culture plate (∼5 × 10^4^ cells in 0.5 mL medium/well) 48 h prior to infection.

24. Label four tubes for virus dilution. Add 1, 5, 25, or 125 μL of primary lysate to each tube. Bring the total volume in each tube to 125 μL by adding PBS.
25. Prior to infection, remove the medium from each well. Replace with 200 μL fresh cell culture medium.
26. Add 125 μL of diluted (or undiluted) virus to each of four wells. Gently pipet up and down to mix. Incubate for 24 h.
27. Carefully remove the medium and wash the monolayers once with 0.5 mL PBS.
28. Fix the cells with 200 μL 4% paraformaldehyde in PBS solution for 10 min.
29. Wash twice with 0.5 mL PBS.
30. Permeabilize the cells with 0.5 mL 0.125% Triton-X100 in PBS for 10 min at room temperature.
31. Wash twice with 0.5 mL PBS.
32. Block with 2% BSA in PBS solution for 30 min.
33. Incubate the cells with a FITC-conjugated antibody against the Ad5 hexon protein for 1 h at room temperature.
  a. Dilute the antibody (1:100) in 2% BSA/PBS solution.
  b. Add 100 μL diluted antibody/well.
34. Carefully remove antibody solution and wash cells once with 0.5 mL PBS.
35. Visualize and count the stained cells for at least three separate fields per well under fluorescence.
36. Determine the average number of positive cells per well. Multiply by the dilution factor to obtain an approximate value of Infectious units (IU)/mL.

***Note:*** We recommend the EVOS Cell Imaging System, which has an automated counting mode that is useful for the assessment of viral titer. The Ad5 reference material from ATCC is the ideal standard.

***Alternatives:*** The QuickTiter Adenovirus Titer Immunoassay Kit can be used for quantification in place of immunofluorescence. Adenovirus titers can be determined with greater precision by the traditional plaque-forming assay (Baer and Kehn-Hall, 2014), or by an end-point dilution method combined with quantitative PCR (Lock et al., 2019).

## Expected outcomes

Robust packaging typically results in acidification of the growth medium and partial detachment of the cell monolayer (Figure 3). These cytopathic effects may be pronounced if the vector design preserves the viral E1 region. Conversely if the E1 region is replaced with transgenic elements, as is often the case, the visible effects may be more subtle or even imperceptible. Regardless of whether the vector retains E1 genes, the concentration of virus should dramatically increase upon amplification.

The yield of virus in the primary lysate (step 17) will vary from vector to vector, but most assemblies should directly produce 10^7^–10^8^ infectious units (IU). Each successive round of amplification should result in a 10–100-fold increase in virus.

## Limitations

The AdenoBuilder protocol is designed for the design and assembly of HAd5-based vectors that feature the deletion of the E1, E3 and/or E4 gene clusters. The generation of high-capacity vectors, including vectors with deletions in E2 or so-called “gut-less” vectors that eliminate all endogenous viral sequences, requires the use of specialized packaging systems. It has not yet been determined if the AdenoBuilder system and methodology can be adapted for such applications.

## Troubleshooting

### Problem 1

The vectors lack compatible restriction sites for a desired vector design. (Before you begin: design a viral vector genome.)

### Potential solution

We have included several unique sites to facilitate directional cloning of restriction fragments (Figure 2). However, some insert sequences or other design elements may preclude the use of some or all of these sites. Fortunately, the compact size of the AdenoBuilder plasmids makes them amenable to alternative cloning approaches that do not require restriction enzyme digestion. A common approach involves the use of high-fidelity PCR enzymes to amplify the entire plasmid, so that the desired insertion site is defined by customized primers (Zeng et al., 2017). The inclusion of overlapping sequences allows the linear PCR product to be circularized by the modified Gibson assembly or Golden Gate assembly prior to bacterial transformation. These cloning strategies are demonstrated in the original AdenoBuilder publication (Miciak et al., 2018).

### Problem 2

The DNA yield of plasmid pAd5-B1 is low. (Before you begin: Select and procure a panel of plasmids for modification and assembly.)

### Potential solution

We find that bacterial cultures that harbor the pAd5-B1 plasmid consistently grow more slowly than the other AdenoBuilder plasmids. We presume that this growth inhibition reflects bacterial toxicity caused by the viral E1 region, as the pAd5-B1 ΔE1-MCS plasmid grows normally. To circumvent this issue, we recommend starting pAd5-B1 preparations from a larger culture volume, as if this were a low-copy plasmid. All of the AdenoBuilder plasmids, including pAd5-B1, were constructed on the pJET1.2 vector backbone, which contains a high-copy number ColE1/pUC origin of replication.

### Problem 3

No cytopathic effects are observed 5 d after electroporation (step 12).

### Potential solution

Cytopathic effects can be subtle, particularly if the desired vector lacks the E1 region. We recommend proceeding with the viral harvest on day 5 regardless of whether cytopathic effects are apparent.

### Problem 4

No virus is evident in the primary lysate (step 36).

### Potential solution

Do not rely on the appearance of the cell monolayer prior to harvest as the sole indicator of virus production. The focus-formation assay (steps 24–36) is considerably more sensitive. If the focus-formation assay fails to detect virus in the primary lysate, troubleshoot as follows.

Review the vector design to ensure that each of the original seven segments is represented in the final assembly. Note that mutants generated in HAd5 genes outside the indicated E1 and E3 regions (Figure 1) may in some cases be functionally complemented by the co-delivery of an undigested plasmid with wild type sequences, as demonstrated in Miciak et al. (2018). In other cases, a bespoke packaging cell line may be required.

Confirm that the level of CO_2_ in the incubator is 5%. We have found that incubators require periodic calibration, and recommend the Fyrite Classic Combustion Analyzer for this purpose.

**Figure 4:**
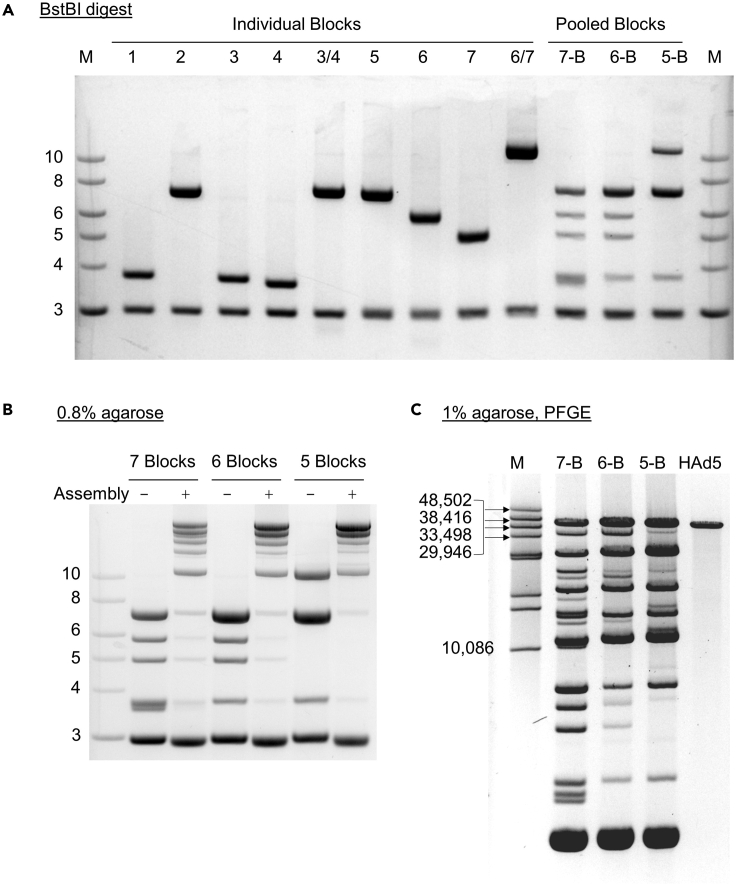
Troubleshooting genome assembly (A) Purified plasmid DNAs should have an OD 260/280 nm ratio between 1.7 and 1.9, and digest completely with BstBI (A). (B and C) Molecular size markers are in kb. Whether the vector design calls for a 7-, 6- or 5- Block assembly (indicated as 7-B, 6-B and 5-B, respectively) each of the 7 basic segments must be included. Assembly (step 3), should result in the appearance of high molecular weight DNAs (B and C). Smaller DNAs corresponding to unligated Blocks are usually apparent, but the bulk of the fragment population should be >10 kb. As visualized by Pulsed field gel electrophoresis (PFGE) the topmost band in the assembly comigrates with genomic DNA harvested from infectious HAd5 (C). Molecular size markers are in bp. Note that 7-Block assemblies typically yield slightly lower amounts of full-length product.

Confirm the identity of each plasmid and the activity of BstBI. First, digest each plasmid with BstBI for 30 min at 65°C then visualize the insert and vector bands on an agarose gel, as shown (Figure 4). If digestion appears to be incomplete, obtain a fresh vial of BstBI (this is unusual).

If there is any ambiguity regarding the insert sizes, the overlapping ends of the plasmid inserts can be confirmed by Sanger sequencing, using the standard primers pJET1.2-forward and pJET1.2-reverse. All plasmid sequences are provided on the Addgene website.

Run an analytical assembly reaction to confirm that digested DNAs are recovered in the ethanol precipitation step (step 2) and full-length genomes are being produced. Add gel loading dye to the DNA assembled in step 3 and analyze on an agarose gel (Figure 4).

***Note:*** We do not find it necessary to routinely assess the extent of DNA assembly. These troubleshooting steps should be performed only if an attempt at packaging has not yielded infectious virus.

### Problem 5

The titer obtained through anti-hexon immunofluorescence differs from the physical titer determined via spectroscopy or qPCR (steps 24–36).

### Potential solution

The detection of hexon protein by immunofluorescence is dependent on viral infection and viral gene expression, and therefore represents a functional titer, sometimes referred to as the infectious titer. In practice, viral packaging inevitably produces many viral particles that are non-functional. Therefore, physical methods used to quantify viruses on the basis of particle density and/or the concentration of viral DNA will result in higher estimates. A combination of physical and functional methods provides the most comprehensive assessment of adenovirus preparations (Lock et al., 2019).

## Resource availability

### Lead contact

Further information and requests for resources and reagents should be directed to and will be fulfilled by the lead contact, Fred Bunz, fredbunz@jh.edu

### Materials availability

AdenoBuilder plasmids can be obtained by academic institutions and nonprofits from Addgene. The identifying codes are included in the supplementary methods table above. https://www.addgene.org/

## Data Availability

This study did not generate code or analyze datasets.
